# Renal Artery Stenosis Predicts Coronary Artery Disease in Patients with Hypertension

**DOI:** 10.1371/journal.pone.0058635

**Published:** 2013-03-14

**Authors:** Thiago A. Macedo, Rodrigo P. Pedrosa, Valeria Costa-Hong, Luiz J. Kajita, Gustavo R. Morais, Jose J. G. De Lima, Luciano F. Drager, Luiz A. Bortolotto

**Affiliations:** 1 Hypertension Unit, Cardiology Division, Heart Institute (InCor) do Hospital das Clínicas da Faculdade de Medicina da Universidade de São Paulo, São Paulo, Brazil; 2 Hemodynamic Laboratory, Cardiology Division, Heart Institute (InCor) do Hospital das Clínicas da Faculdade de Medicina da Universidade de São Paulo, São Paulo, Brazil; Brigham and Women's Hospital, Harvard Medical School, United States of America

## Abstract

In hypertensive patients with indication of renal arteriography to investigate renal artery stenosis (RAS) there are no recommendations regarding when to investigate coronary artery disease (CAD). Moreover, the predictors of CAD in patients with RAS are not clear. We aimed to evaluate the frequency and the determinants of CAD in hypertensive patients referred to renal angiography. Eighty-two consecutive patients with high clinical risk suggesting the presence of RAS systematically underwent renal angiography and coronary angiography during the same procedure. Significant arterial stenosis was defined by an obstruction≥70% to both renal and coronary territories. Significant CAD was present in 32/82 (39%) and significant RAS in 32/82 (39%) patients. Both CAD and RAS were present in 25.6% from the 82 patients. Patients with severe CAD were older (63±12 vs. 56±13 years; *p = 0.03*) and had more angina (41 vs. 16%; *p = 0.013*) compared to patients without severe CAD. Significant RAS was associated with an increased frequency of severe CAD compared to patients without significant RAS (66% vs. 22%, respectively; *p<0.001*). Myocardial scintigraphy showed ischemia in 21.8% of the patients with CAD. Binary logistic regression analysis showed that RAS≥70% was independently associated with CAD≥70% (OR: 11.48; 95% CI 3.2–40.2; *p<0.001*), even in patients without angina (OR: 13.48; 95%CI 2.6–12.1; *p<0.001*). Even considering a small number of patients with significant RAS, we conclude that in hypertensive patients referred to renal angiography, RAS≥70% may be a strong predictor of severe CAD, independently of angina, and dual investigation should be considered.

## Introduction

Atherosclerotic renal artery stenosis (RAS) is a well-recognized cause of secondary arterial hypertension and independently associated with cardiovascular events [1], [2]. Previous studies have shown coexistence of RAS with atherosclerosis in other sites [3], [4] and an elevated prevalence of RAS in patients with coronary artery disease (CAD) diagnosed by cardiac catheterization [5], [6]. More than a common association, the presence of RAS seems to be equivalent to coronary artery disease in terms of cardiovascular risk [5]. Regarding the increased prevalence of RAS among patients with CAD and the poorer prognosis associated with the presence of RAS the American Heart Association/American College of Cardiology recommends performing renal angiography at the same time as coronary angiography, when the patient with CAD has unexplained renal failure, resistant hypertension or multivessel coronary disease [7]. However, it is not clear when to perform coronary angiography in hypertensive patients referred for renal angiography with suspicious of RAS. Indeed, in the 2011 European Society of Cardiology Guidelines on The Diagnosis and Treatment of Peripheral Artery Diseases and 2011 American College of Cardiology Foundation/American Heart Association Focused Update of The Guideline for The Management of Patients with Peripheral Artery Disease [8], [9], there are no recommendations regarding when to investigate CAD in patients with RAS. Thus, the purpose of our study was to evaluate the frequency of CAD in patients with hypertension referred for renal angiography and to analyze potential predictors of this association. We hypothesize that the presence of significant RAS could be a useful marker to identify CAD in hypertensive patients referred for renal angiography.

## Methods

We evaluated consecutive patients with established hypertension and suspicion of RAS based on clinical data (resistant hypertension, hypertensive pulmonary edema, congestive heart failure, malignant hypertension, or progressive renal failure) referred to the Hypertension Unit of the Heart Institute (InCor) from February 2009 to February 2011. The procedures were carried out in accordance with the institutional guidelines and the institutional review committee approved the protocol (1125/07 – Ethics Committee for Analysis of Research Projects HC-FMUSP). All patients gave informed consent in writing to participate in the study.

Patients were selected according to the risk of RAS based on clinical data and additional diagnostic noninvasive imaging suggesting the presence of RAS: (1) decreased renal perfusion by renal scintigraphy; (2) increased renal blood flow velocity (≥180 cm/s) observed at Doppler ultrasound of renal arteries and/or (3) magnetic resonance angiography of renal arteries suggesting stenosis, whose angiography would be useful to the diagnosis of significant RAS. We excluded patients with known CAD (prior myocardial infarction, coronary artery revascularization, and previous ischemic acute syndrome), stable angina [Canadian Cardiovascular Society III–IV], unstable angina) and RAS detected by prior renal angiography.

Fasting blood samples were obtained for the measurement of total cholesterol, low-density lipoprotein cholesterol, high-density lipoprotein cholesterol, and serum creatinine. The diagnosis of hypercholesterolemia was considered if the patients had been prescribed cholesterol-lowering agents or they had fasting total cholesterol >200 mg/dL. Patients were considered to have *diabetes mellitus* if dietary or pharmacological interventions were required to maintain normal blood glucose levels (<126 mg/dL) or under use of specific medications. Anthropometric data included body mass index (BMI) in kg/m2 as well as waist and hip circumferences. The blood pressure (BP) was measured after five minutes resting using an automatic digital sphygmomanometer (OMRON-705CP, Japan). The mean of two readings was used in the analysis.

### Evaluation of the coronary and renal arteries

After undergoing a complete clinical examination, including a detailed medical history, all the patients underwent renal and coronary angiography in the same setting and renal procedure was performed by panoramic and selective injection into the renal arteries. Coronary artery disease was defined as lesions with≥70% arterial obstruction in at least one epicardial coronary artery, according to the visual analysis of a blinded examiner for renal artery condition. For better quantification of coronary stenosis, also a Quantitative Coronary Angiography (QCA) [10] was performed when lesions are not correctly identified by visual analysis. In this system, the vessel diameter is calculated in absolute values (mm) by detecting the boundaries of a section of the contrast catheter and comparing the computed mean catheter diameter. The reference vessel diameter is based on the computer estimation of the original arterial dimensions at the stenosis site. For a statistical comparison of clinical and laboratory variables, the study population was divided into 2 groups according to the presence (≥70%) or absence (<70%) of severe coronary obstruction verified by coronary angiography. In patients who had a diagnosis of severe CAD after the coronary angiography, we performed myocardial perfusion scintigraphy to evaluate the presence of related myocardial ischemia.

Serum creatinine was determined twice during the evaluation: 1 day before the procedure and 3 days after. Glomerular filtration rate (GFR) was estimated by MDRD formula (Modification of Diet in Renal Disease) [11]. For prevention of nephropathy induced by iodinated contrast, patients who had estimated GFR (eGRF) below 60 mL/min/m^2^received normal saline infusion (1 mL/kg/h) before and 24 h after the procedure plus 600 mg oral *N*-acetyl-cysteine twice daily, and low-osmolar contrast for the procedure, as previously described [12].

### Statistical analysis

Data were analyzed with SPSS 17.0 statistical software. Descriptive analysis was used to define the study population. Results of parametric data were expressed as mean±standard deviation and nonparametric as median, followed by interquartile range or percentages, when appropriate. The Student *t* test for independent samples and Mann-Whitney U were used to compare quantitative variables of groups with and without CAD. Chi-square test was used for qualitative variables, and Fisher correction was used when necessary. Univariate and multiple logistic regression analysis were used to determine the factors associated with severe CAD in the entire population. Variables with a p value<0.1 in univariate analysis were used in multivariate models. A p value <0.05 was considered significant.

## Results

**Figure 1:**
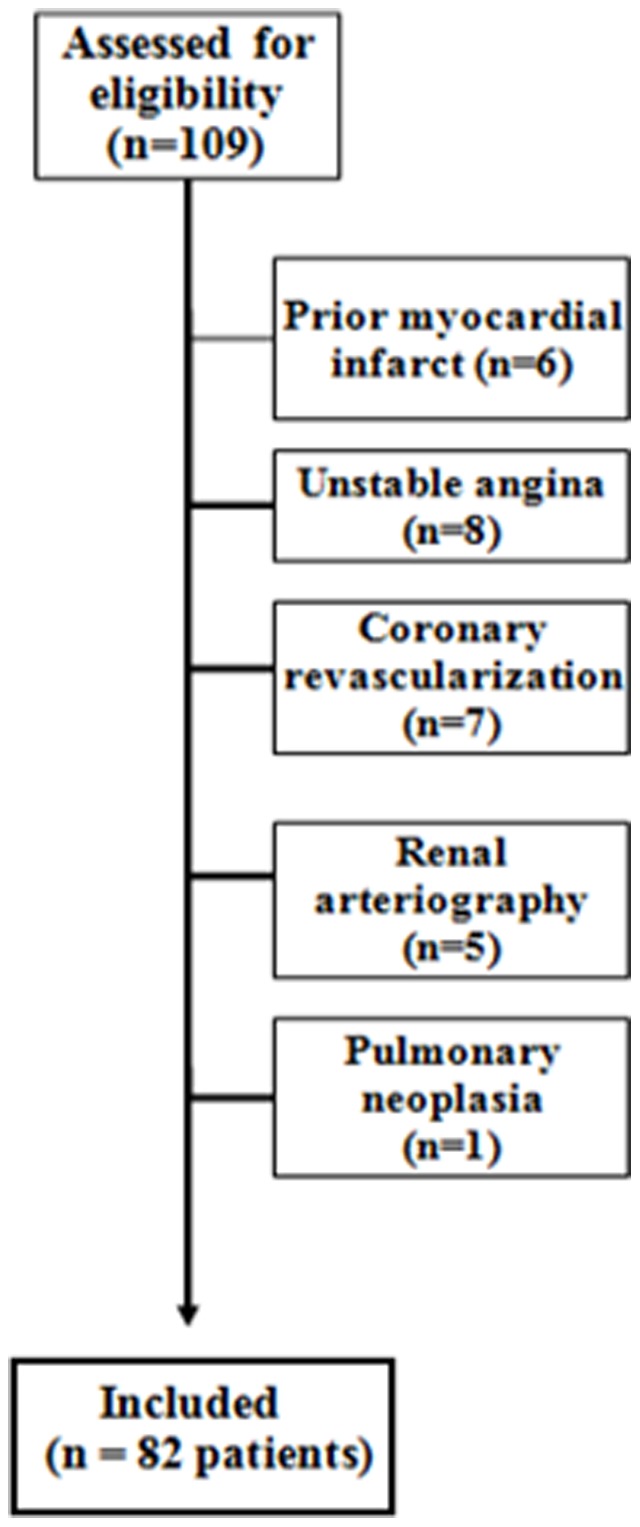
Patient selection flowchart.

We initially included 109 consecutive patients with suspicion of RAS. Twenty-one patients with known CAD in prior coronary angiography, five patients with known renal atherosclerosis (from previous renal angiography), and one with respiratory instability were excluded (Figure 1). Thus, 82 patients were included in the final analysis. Significant RAS was present in 32/82 (39%) and severe CAD present in 32/82 (39%) patients. There was concordance of significant RAS and CAD in 25.6% from the 82 patients. The average of contrast volume used to renal was 110 ml. Only 7 (8.5%) patients had a transient and mild/moderate increase in creatinine levels suggestive of contrast-induced nephropathy. Dialysis was not required and none developed serious complications during both procedures at the same setting.

The main characteristics of the total sample and according to the presence or absence of severe CAD are shown in Table 1. We observed a high percentage of associated diabetes, claudication and smoking. In comparison with patients without severe CAD, the patients with severe CAD were older, had more symptoms of angina and had a higher percentage of significant RAS (Table 1). There were no significant differences between the 2 groups concerning risk factors, such as diabetes, smoking status, claudication, blood pressure, and dyslipidemia. Significant RAS was associated with an increased prevalence of severe CAD compared to patients without significant RAS (66% vs. 22%, respectively; *p<0.001*). Myocardial scintigraphy was performed in ninety-one percent of the patients with CAD (regardless the presence of angina). Of them, myocardial ischemia was documented in 21.8% of the patients. We found 37.5% of myocardial ischemia in the group of patients with angina and only 3.5% in patients without angina. No differences in the glomerular filtration rate (eGRF) were observed in patients with and without RAS [45.4 (39.7−51.0)×47.7 (41.9−53.5); p = 0.30)].

**Table 1:** Clinical characteristics of the 82 patients according to the presence of coronary artery disease.

Variables	Total (N = 82)	CAD (<70%) (N = 50)	CAD (≥70%) (N = 32)	p value
Age, years	59±13	56±13	63±12	0.03*
Male, %	34	34	44	0.38
Caucasoid, %	26	22	31	0.54
Diabetes mellitus, %	44	42	47	0.66
Smoking, %	32	30	34	0.67
Angina, %	26	16	41	0.013*
SBP, mmHg	145±28	141±29	151±27	0.14
PP, mmHg	67.0 (52.5−85.0)	60.0 (50.0−85.2)	72.0 (61.7−85.2)	0.07
MAP, mmHg	99.5±16.1	97.3±15.4	103.1±17.8	0.12
Antihypertensives, n	4 (3−5)	4.5 (4−5)	4 (3−5)	0.19
BMI, kg/m^2^	29.5±6.4	30.0±6.5	28.7±6.2	0.30
Serum creatinine, mg/dL	1.44 (1.1−2.3)	1.38 (1.00−1.89)	1.59 (1.11−2.76)	0.08
eGFR<60 ml/min/m^2^, %	63	56	75	0.08
LDL, mg/dL	110±35	107±33	114±39	0.40
HDL, mg/dL	40 (35−48)	41 (36−48)	37 (32−44)	0.08
Triglycerides, mg/dL	134 (95−178)	130 (90−170)	154 (98−234)	0.15

CAD  =  coronary artery disease; SBP  =  systolic blood pressure; PP  =  pulse-pressure pressure; MAP  =  mean arterial pressure; BMI  =  body mass index; eGFR  =  estimated glomerular filtration rate; LDL  =  low-density lipoprotein cholesterol; HDL  =  high-density lipoprotein cholesterol.

In the univariate analysis, the variables significantly correlated with the presence of severe CAD were age, angina, eGFR, triglycerides, and RAS≥70% (Table 2). Among the 50 patients without RAS, 12 (22%) had CAD and, in this group, the presence of angina was the only predictor of CAD (OR: 6.45, 95% CI: 1.29–32.15, *p<0.001*).The 95% CI round proportions of the presence of significant CAD or RAS, or both, were expressed in the Table 3. In the multivariate logistic regression analysis, angina (OR: 6.56, 95% CI: 1.70–25.30, *p = 0.006*) and RAS≥70% (OR: 11.48, 95% CI: 3.26–40.25, *p<0.001*) were the only independent variables associated with severe CAD. Renal artery stenosis was a strong predictor even in the subgroup of patients without angina (OR: 13.48; 95%CI 2.6–12.1; *p<0.001*).

**Table 2:** Unadjusted and adjusted odds ratios variables in patients referred to renal angiography according to severe coronary artery disease.

	Univariate			Multivariate		
Variables	OR (CI)	p value	β	SE	OR (CI)	p value
Age	1.04 (1.00−1.08)	0.03				
eGFR	2.36 (0.89−6.25)	0.09				
HDL	0.97 (0.93−1.01)	0.13				
Triglycerides	1.01 (1.00−1.01)	0.06				
Angina	3.60 (1.28−10.10)	0.015	1.88	0.69	6.56 (1.70−25.30)	0.006*
RAS ≥70%	6.77 (2.52−18.21)	<0.001	2.44	0.64	11.48 (3.26−40.25)	<0.001*

OR  =  odds ratio; eGRF  =  estimated glomerular rhythm of filtration; CI  =  confidence interval; HDL  =  high-density lipoprotein cholesterol; RAS  =  renal artery stenosis.

**Table 3:** Percentage of patients with or without significant CAD and RAS and the respective 95% CI round proportions.

	CAD	No CAD
**RAS**	**21 patients (CI: ± 9.4) [16.1% to 35.0%]**	**11 patients (CI: ±7.4) [6.0% to 20.8%]**
**No RAS**	**11 patients (CI: ±7.4) [6.0% to 20.8%]**	**39 patients (CI: ± 10.8) [36.7% to 58.3%]**

CI  =  confidence interval; CAD  =  coronary artery disease ≥ 70%; RAS  =  renal artery stenosis ≥ 70%.

## Discussion

To the best of our knowledge, the present investigation has several new findings: (1) In hypertensive patients referred to renal angiography because of significant risk for RAS, the frequency of severe CAD was 39%; (2) In the presence of significant RAS, CAD≥70% was highly prevalent (66%) and renal and coronary angiography performed at the same time did not significantly increase the rate of complications associated with the procedures; (3) RAS≥70%, diagnosed by renal angiography, was a strong and useful predictor to identify severe CAD.

Atherosclerotic RAS is a clinical condition frequently observed with multiple cardiovascular risk factors and atherosclerosis in another arterial beds [13], [14], especially in patients with coronary artery disease [15]. Several reports have demonstrated a high frequency of RAS in patients with CAD during routine cardiac catheterization. Moreover, a positive association between the number of affected coronary vessels and RAS prevalence has been reported [4], [16]. However, this evidence cannot be extrapolated to patients with hypertension referred to renal angiography for investigation of RAS. Since atherosclerosis is a systemic disease, it is conceivable that a significant proportion of hypertensive patients who have confirmed RAS also have CAD. However, evidence in this important research area is scanty. To the best of our knowledge, there is one single study also from Brazil involving a small number of patients (n = 23) reporting a high prevalence of CAD (74%) in patients with significant RAS [17]. It should be stressed that this preliminary investigation did not systematically evaluate patients at risk of RAS, and the definition of CAD also included patients with less severe coronary stenosis (>50%). In addition, they did not explore predictors of CAD in patients with RAS. We studied the prevalence of CAD in the group without or with non-significant RAS. In this subgroup, angina was the only predictor of CAD, suggesting that this traditional symptom should guide the CAD diagnosis. Therefore, our results point out that asymptomatic patient also had significant stenosis in coronary arteries, so that the indication for coronary angiography based only on the presence of symptoms could neglect a significant number (22%) of patients at high risk for CAD. Although we actively searched only for anatomical CAD, non-invasive indices of myocardial ischemia were assessed as a potential diagnostic tool to improve risk stratification. We found myocardial ischemia in 21.8% of the patients and new interventions (not scheduled) were performed. Twenty-five percent (8/32) of the patients with severe CAD underwent surgical revascularization (4) and percutaneous treatment (4). Although our results do not support the concept that is obligatory performing coronary angiography at the same setting in hypertensive patients with RAS≥70%, the present study suggests that hypertensive patients with RAS≥70% deserve additional investigation for CAD, even using non-invasive techniques.

In our study, the renal function estimated by eGFR calculated by using the MDRD formula was also correlated to the presence of significant CAD. Currently, eGFR has been considered an important predictor of cardiovascular events in larger populations [11] and the alone presence of severe CAD diagnosed by coronary angiography is a predictor of cardiac events independent of noninvasive evaluation, especially in patients with chronic kidney disease [18]. The majority of our patients had some degree of renal dysfunction, and this fact could be implicated in the association between RAS and CAD. This finding was also consistent with previous studies that demonstrated that RAS is an important factor in progressive renal and cardiac diseases and increased mortality [1], [19].

The present investigation has some limitations that should be addressed. First of all, our sample size was small mainly explained by our single-center study design investigated only patients with high clinical risk suggesting the presence of RAS. Second, the selection criteria for indication of renal angiography were based on clinical features of renovascular hypertension, and noninvasive procedures such as renal cintigraphy, Doppler ultrasound, and magnetic resonance of renal arteries. It is well known that in patients with a reduced glomerular filtration rate, renal scintigraphy has a low diagnostic value, and magnetic resonance angiography has a tendency toward overestimation of stenosis [20]. Third, the selection of hypertensive patients with high risk for atherosclerotic RAS could overestimate the number of individuals affected by atherosclerosis in others vessels including coronary arteries. However, both limitations are related to the diagnostic procedures and standard indications for investigating RAS *per se* and could partially explain the low rate of RAS in selected patients and the higher rate of severe CAD (66%) in patients with significant RAS, respectively.

In conclusion, our data suggest that significant RAS (≥70%), when diagnosed in patients referred for renal angiography, is a useful predictor to the presence of severe CAD. We observed a close association between significant RAS and severe CAD in hypertensive patients. Since a non-negligible percentage of these patients had myocardial ischemia, we conclude that in hypertensive patients referred to renal angiography, RAS≥70% may be a strong predictor of severe CAD, independently of angina, and dual investigation should be considered.
